# The antigenic identity of human class I MHC phosphopeptides is critically dependent upon phosphorylation status

**DOI:** 10.18632/oncotarget.16952

**Published:** 2017-04-08

**Authors:** Fiyaz Mohammed, Daniel H. Stones, Angela L. Zarling, Carrie R. Willcox, Jeffrey Shabanowitz, Kara L. Cummings, Donald F. Hunt, Mark Cobbold, Victor H. Engelhard, Benjamin E. Willcox

**Affiliations:** ^1^ Cancer Immunology and Immunotherapy Centre, Institute of Immunology and Immunotherapy, University of Birmingham, Edgbaston, Birmingham B15 2TT, UK; ^2^ Carter Immunology Center and Department of Microbiology, University of Virginia School of Medicine, Charlottesville, Virginia 22908, USA; ^3^ Department of Chemistry, University of Virginia, Charlottesville, Virginia 22908, USA; ^4^ School of Immunity and Infection, University of Birmingham, Edgbaston, Birmingham B15 2TT, UK; ^5^ Current address: Cancer Centre, Massachusetts General Hospital, Boston, Massachusetts 02114, USA; ^6^ Current address: Department of Medicine, Harvard Medical School, Charlestown, Massachusetts 02129, USA

**Keywords:** tumour immunology, phosphopeptide, peptide-MHC complex, neoepitope, peptide conformation

## Abstract

Dysregulated post-translational modification provides a source of altered self-antigens that can stimulate immune responses in autoimmunity, inflammation, and cancer. In recent years, phosphorylated peptides have emerged as a group of tumour-associated antigens presented by MHC molecules and recognised by T cells, and represent promising candidates for cancer immunotherapy. However, the impact of phosphorylation on the antigenic identity of phosphopeptide epitopes is unclear. Here we examined this by determining structures of MHC-bound phosphopeptides bearing canonical position 4-phosphorylations in the presence and absence of their phosphate moiety, and examining phosphopeptide recognition by the T cell receptor (TCR). Strikingly, two peptides exhibited major conformational changes upon phosphorylation, involving a similar molecular mechanism, which focussed changes on the central peptide region most critical for T cell recognition. In contrast, a third epitope displayed little conformational alteration upon phosphorylation. In addition, binding studies demonstrated TCR interaction with an MHC-bound phosphopeptide was both epitope-specific and absolutely dependent upon phosphorylation status. These results highlight the critical influence of phosphorylation on the antigenic identity of naturally processed class I MHC epitopes. In doing so they provide a molecular framework for understanding phosphopeptide-specific immune responses, and have implications for the development of phosphopeptide antigen-specific cancer immunotherapy approaches.

## INTRODUCTION

MHC-restricted phosphopeptides represent promising tumour-associated antigens for cancer immunotherapy. Phosphopeptide antigens are naturally processed and presented on human tumour cells by class I and class II MHC molecules [1–5]. Consistent with the prevalence of phosphorylation, phosphopeptides are presented by multiple MHC molecules [1, 4, 5], and may comprise a substantial portion of the peptide repertoire for some alleles. Initial mass spectrometric analysis of phosphopeptide presentation highlighted different patterns of expression on separate tumour cell lines [1, 5], suggesting distinct tumour-specific immunological signatures of “transformed self”. Furthermore, phosphopeptide-specific T cells can recognize intact human tumour cells [1, 4, 5], highlighting their therapeutic potential. Moreover, phosphopeptide antigens may be closely linked to maintenance of the malignant phenotype. Dysregulated protein kinase activity, normally tightly controlled, is a hallmark of malignant transformation, and contributes directly to oncogenic signalling pathways leading to uncontrolled proliferation, cell survival, tissue invasion and metastasis [6–10]. Secondly, the source proteins for phosphopeptide antigens include those involved in cytoplasmic signalling pathways, metabolism, or cell cycle regulation, many of which are implicated in cellular transformation [1, 4, 5]. Consistent with this, we recently identified numerous phosphopeptide antigens selectively presented on primary haematological malignancies, many of which were immunogenic and might contribute to tumour immunosurveillance [11]. These factors suggest phosphopeptide antigens may provide functionally important targets for cancer immunotherapy.

Despite their potential clinical relevance, the influence of phosphorylation on the antigenic identity of naturally occurring phosphopeptides is currently unclear. One possibility, supported by recent structural data [12], is that the conformation of MHC-bound phosphopeptide epitopes closely matches that of their unmodified counterparts. In this case phosphopeptide-specific immunotherapy strategies such as TCR gene transfer would ideally focus not only on the specific antigenic peptide target, but also significantly on the phosphate moiety itself. A second possibility is that phosphorylation might profoundly influence the MHC-bound phosphopeptide conformation, creating conformationally novel antigens. This could increase therapeutic targeting options, which include vaccination and adoptive T cell transfer approaches [13, 14]. However such major conformational changes have not been demonstrated to date. Finally, although phosphopeptide recognition by T cells *in vitro* is observed to be both epitope-specific and phosphate-dependent [1, 4, 5], molecular evidence establishing the extent to which this is TCR-dependent, and how complete discrimination is at the TCR level, is lacking.

Although our previous studies on class I MHC phosphopeptide presentation did not address these issues, they established that the phosphate group can strongly influence peptide-MHC (pMHC) interaction [15]. We defined a canonical motif, involving a phosphorylation at position 4 and a positively charged side chain at position 1 (R/K), accounting for ˜ 50% of the HLA-A2 phosphopeptide repertoire. This motif was frequently associated with subdominant anchor residues. For phosphopeptides with this canonical motif (hereafter referred to as canonical phosphopeptides), the phosphate moiety could act as a “phosphate surface anchor”, mediating extensive contacts to HLA-A2. Furthermore, phosphate-mediated contacts were highly energetically favourable and could compensate for suboptimal anchor residue interactions. These findings raised the possibility that phosphorylation might exert a major influence on both peptide conformation and TCR recognition.

To help resolve these issues, we solved structures of three canonical phosphopeptide-HLA-A2 complexes and their non-phosphorylated counterparts, and assessed the effects of phosphorylation on pMHC affinity. Also, we studied the ability of the TCR to discriminate phosphorylated from unmodified forms of the same naturally occurring epitope, using a soluble TCR from a functionally phosphopeptide-specific T cell clone. Our results suggest phosphorylation can exert a critical influence on both epitope conformation and TCR/pMHC binding, and highlight the possibility of targeting specific tumour associated phosphopeptides for cancer immunotherapy.

## RESULTS

### Assessing the structural effect of phosphorylation on three HLA-A2-bound phosphopeptides

**Figure 1:**
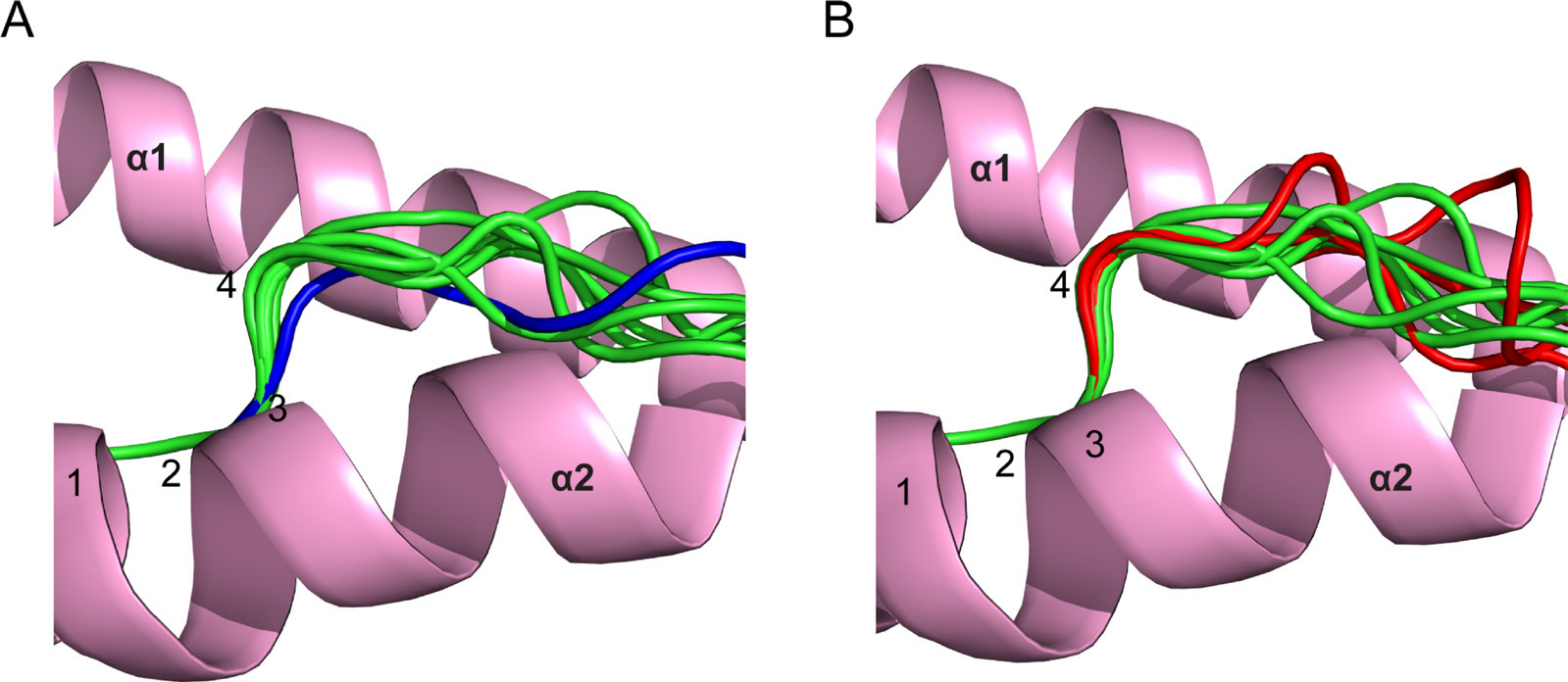
Analysis of main chain conformations of phosphopeptides (**A**) Comparison of peptide main-chain conformation around position 4 for a canonical phosphopeptide derived from insulin receptor substrate 2 (coloured blue; PDB code 3FQX obtained from a previous study by Petersen *et al*. [12]), relative to other canonical phosphopeptide structures from previous studies [12, 15] (green). (**B**) Comparison of main chain conformations of PKD2p, RQA_Vp, and RQIp (all shown in red), relative to previously solved canonical phosphopeptides (shown in green). All superpositions were based on Cα atoms of residues at positions 1–3.

Previously, we noted that canonical phosphopeptides exhibited a restrained main chain conformation around the position 4 Cα compared to non-phosphorylated peptides [15]. Although consistent with phosphorylation-induced conformational changes, this analysis did not compare the same peptides in their phosphorylated/non-phosphorylated states, and an alternative explanation was that canonical phosphopeptides are restricted to those that naturally adopt this restrained conformation in the unmodified state. The only study to make a direct comparison [12] established that a single canonical motif phosphopeptide did not alter in conformation in the unmodified state. However, the presence of a Proline close to the phosphate in this epitope prevented it adopting the restrained conformation of previously solved canonical phosphopeptides, suggesting it may be atypical of canonical phosphopeptides (Figure 1A).

**Figure 2:**
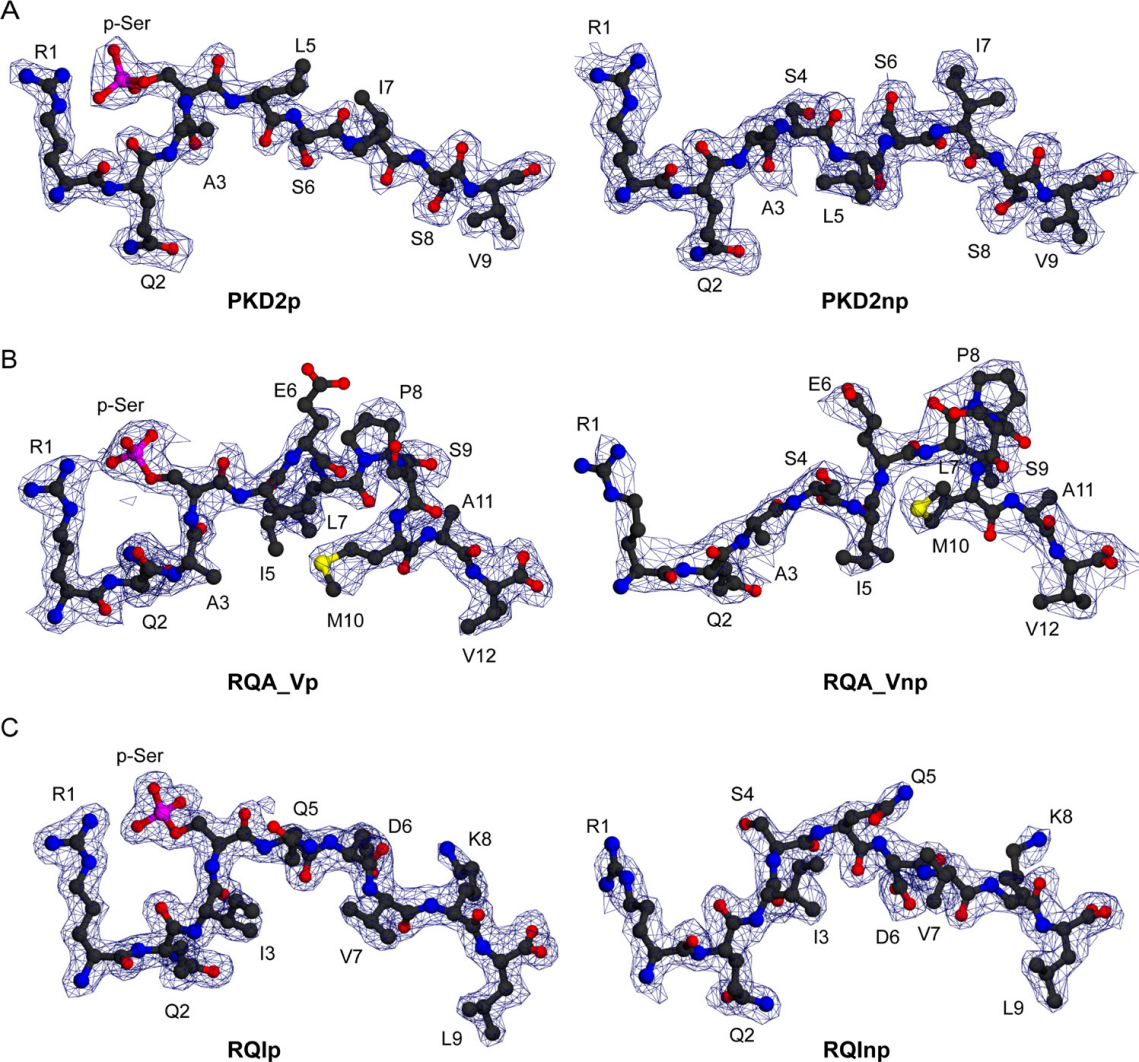
Electron density for three HLA-A2 bound phosphopeptides in phosphorylated and unmodified states (**A**) Structure of PKD2p (RQApSLSISV) and PKD2np (RQASLSISV) (left and right, respectively), each superimposed on a *2Fo-Fc* electron density map contoured at 1.0 σ (blue wire). (**B**) Structure of RQA_Vp (RQApSIELPSMAV) and RQA_Vnp (RQASIELPSMAV) (left and right, respectively), pictured as in A. (**C**) Structure of RQIp (RQIpSQDVKL) and RQInp (RQISQDVKL) (left and right, respectively), pictured as in A.

To address this, we determined HLA-A2 complex structures of three canonical phosphopeptide antigens (RQApSlSISV, termed PKD2 and derived from Protein Kinase D2; RQApSIELPSMAV, termed RQA_V and derived from Lymphocyte Specific Protein 1 (LSP-1); RQIpSQDVKL, termed RQI and derived from adenosine monophosphate deaminase 2 (AMPD2)) [5] in both phosphorylated and non-phosphorylated states (Supplementary Table 1). We observed well-defined electron density for each peptide moiety (Figure 2). These antigens are attractive therapeutic targets, as PKD2 is dysregulated in several solid tumours, implicated in the transformation process, and a target for chemotherapy [16]; LSP-1 is a marker of lymphoma [17], and the RQA_V epitope is elevated on the surface of a range of tumour cell lines and primary leukemic tissue [11]; finally AMPD2 expression has been noted on both melanoma and ovarian carcinoma cell lines [5]. Unsurprisingly, each epitope structure adopted a restrained main chain conformation at the position 4 Cα in its modified form, typical of previously examined canonical phosphopeptides (Figure 1B). Also, conserved pMHC contacts to the N and C terminus and anchor residues within the B and F pocket were retained for all three epitopes in both unmodified and modified forms.

### A conformational change in PKD2 permits phosphorylation-dependent MHC binding

**Figure 3:**
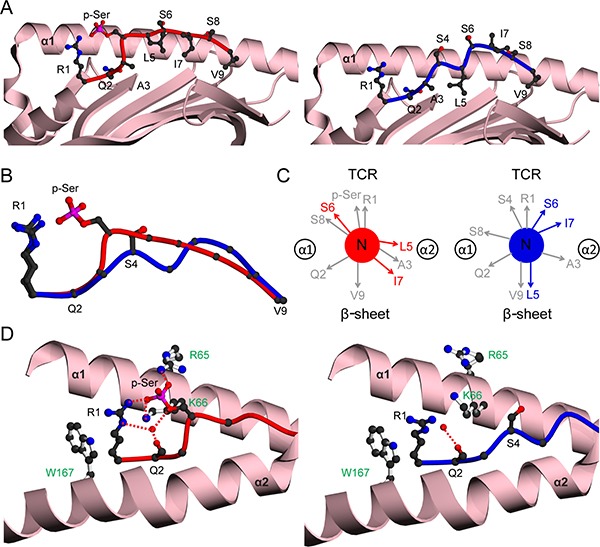
Structural comparison of HLA-A2 bound PKD2 phosphopeptide in modified and unmodified states (**A**) HLA-A2 bound structures of PKD2p (left, red) and PKD2np (right, blue). The α1-α2 antigen binding platform is shown in ribbon representation (pink), with α2 helix residues 137-166 omitted for clarity. (**B**) Superposition of the PKD2 main chain structures for phosphorylated (red) and non-phosphorylated (blue) peptides, including the R1 and S4/p-Ser side-chains. (**C**) Side-chain orientation for phosphorylated (left, red) and non-phosphorylated (right, blue) PKD2 peptides, as viewed along the long axis of the peptide from its N-terminus. Side chains which exhibit substantial changes in orientation upon phosphorylation are highlighted in red. (**D**) Interactions of position 4 side-chains for phosphorylated (left, red main chain) and non-phosphorylated (right, blue main chain) peptides with HLA-A2 α1-α2 helices (pink). HLA-A2 side chains are shown as white sticks and labelled green. Hydrogen bonds are indicated by red dashed lines; the red spheres represent water molecules; for clarity the underlying β-sheet is omitted.

Typical of canonical HLA-A2-restricted phosphopeptides that bear suboptimal anchors, the PKD2 phosphopeptide exhibits phosphate-dependent binding to HLA-A2, with its affinity higher in the phosphorylated state (Kd 38.5 nM vs 284.5 nM for modified and unmodified forms respectively) [15]. Comparisons of HLA-A2-PKD2p and HLA-A2-PKD2np (both in P2_1_2_1_2_1_, Supplementary Table 1) revealed a very similar overall MHC conformation, with an rmsd value of 0.21 Å. In contrast, the same comparisons clearly showed a major change in epitope conformation (Figure 3A–3C), reflected by a much larger peptide rmsd value (1.41Å) than previous analogous comparisons (range 0.09–0.45Å) [12]. In the unmodified PKD2np, S4 adopts a low position in the binding groove (Figure 3B) and forms no contacts to the MHC (Figure 3D, right). Upon phosphorylation the epitope adopts a raised conformation at position 4 enabling additional phosphate-mediated MHC and intra peptide contacts, typical of canonical phosphopeptides (Figure 3B, Figure 3D, left). Most importantly, phosphorylation results in major reorientation of the main chain and side chains at residues 5, 6 and 7 (Figure 3C), positions that frequently contact the TCR. Consequently, the molecular surface presented for T cell recognition by HLA-A2-PKD2p and HLA-A2-PKD2np is substantially different. The conformational change also provides an explanation for the effect of phosphorylation on PKD2 affinity for HLA-A2: although the differing main chain conformations alter pMHC contacts, the net energetic effect is likely minimal, other than the additional p-Ser-mediated MHC contacts, which most probably underlie the stronger HLA-A2-PKD2p interaction.

### The RQA_V epitope undergoes a radical conformational rearrangement upon phosphorylation

**Figure 4:**
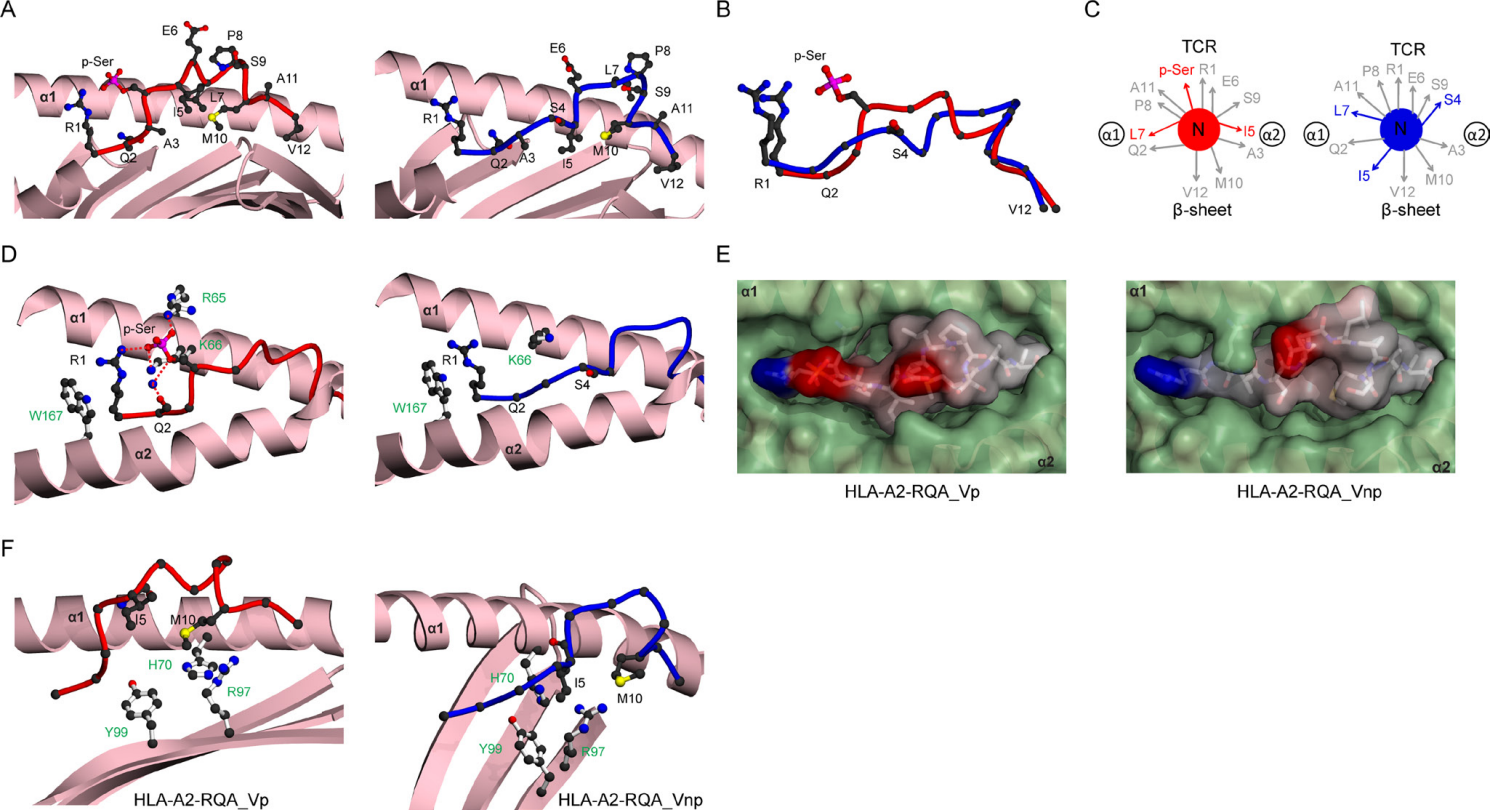
Structural rearrangement of RQA_V phosphopeptide upon phosphorylation (**A**) HLA-A2 bound structures of RQA_Vp (left, red) and RQA_Vnp (right, blue), derived from Lymphocyte specific protein 1. The α1-α2 antigen binding platform is shown in ribbon representation (pink), with α2 helix residues 137-166 omitted for clarity. (**B**) Superposition of the RQA_V main chain structures for phosphorylated (red) and non-phosphorylated (blue) peptides, including the R1 and S4/p-Ser side-chains. (**C**) Side-chain orientation for phosphorylated (left, red) and non-phosphorylated (right, blue) RQA_V peptides, as viewed along the long axis of the peptide from its N-terminus. Side chains which exhibit substantial changes in orientation upon phosphorylation are highlighted in red. (**D**) Interactions of position 4 side-chains for phosphorylated (left, red main chain) and non-phosphorylated (right, blue main chain) peptides with HLA-A2 α1-α2 helices (pink). Hydrogen bonds are indicated by red dashed lines; the blue sphere represents a sodium atom; for clarity the underlying β-sheet is omitted. HLA-A2 side chains are shown as white sticks and labelled green. (**E**) Molecular surface of phosphorylated (left) and non-phosphorylated (right) RQA_V peptides in complex with HLA-A2, as viewed from the perspective of the TCR. The α1-α2 molecular surface is shown in green, whereas the peptide surface is coloured according to electrostatic potential (blue, positive; grey, neutral; red, negative). The potential scale ranges from -7 (red) to +7 (blue) in units of kT/e. (**F**) Altered positioning of I5 in RQA_Vnp (right, blue) allowing additional contacts to HLA-A2 side-chains R97, H70 and Y99, relative to RQA_Vp (left, red), none of which are observed in RQA_Vp. The peptide binding platform is shown in ribbon representation (pink), with the α2 helix omitted for clarity.

We also examined the structure of RQA_V, a 12-residue phosphopeptide [5], in both phosphorylated and non-phosphorylated forms. Although RQA_V possesses the canonical motif and incorporates a subdominant anchor residue at position 2, surprisingly pMHC affinity analyses showed similar binding affinities for modified and unmodified versions (Kd 60 nM and 26 nM respectively). This was particularly intriguing because the overlapping RQA_M epitope, which comprises the first 10 amino acids of the RQA_V epitope, displays highly phosphate-dependent binding to HLA-A2 (Kd 11.2 nM and 1769 nM for modified and unmodified forms, respectively) [15]. We initially hypothesised that the presence of two additional C-terminal amino acids in RQA_V might disrupt phosphate-mediated contacts to the MHC molecule. However, the structure of the phosphorylated form of RQA_V bound to HLA-A2 (HLA-A2-RQA_Vp) at 2.1Å (Supplementary Table 1, Figure 4A–4C) confirmed that phosphate-mediated contacts (Figure 4D, left,) were extremely similar to those of RQA_M, which we previously showed were highly energetically significant [15]. In fact, the additional two residues were accommodated via a novel helical segment towards the C terminus of the phosphopeptide (Figure 4A, left). This suggested an alternative hypothesis, that the non-phosphorylated form of the epitope (RQA_Vnp) underwent a conformational rearrangement such that it was energetically equivalent to that of the phosphorylated version (RQA_Vp) in terms of the strength of pMHC binding.

To test this, we solved the structure of RQA_Vnp in complex with HLA-A2 (HLA-A2-RQA_Vnp). We failed to crystallize the HLA-A2-RQA_Vnp complex using conventional approaches, even in conditions used to crystallise a wide range of HLA-A2 complexes [12]. To circumvent these problems, we co-crystallized the unmodified complex in the presence of LILRB1 (LIR-1, ILT2) [18], a broadly expressed inhibitory receptor that recognises class I MHC with low affinity and which we have previously co-crystallised in complex with HLA-A2 [19]. Crucially, the LILRB1 binding site on HLA-A2 involves the α3 and β2m domains but does not involve the α1-α2 peptide-binding platform [19], and peptides crystallised in HLA-A2 are identical in conformation in the presence or absence of LILRB1 [19, 20]. Crystallisation trials of HLA-A2-RQA_Vnp with LILRB1 yielded LILRB1-HLA-A2-RQA_Vnp complex crystals, allowing the structure to be solved to 2.7Å (Figure 4A, right, Supplementary Table 1).

Comparison of unmodified and modified structures showed the RQA_V epitope undergoes a dramatic change in conformation upon phosphorylation (Figure 4A–4C), greater than for PKD2, reflected in the higher peptide rmsd value (RQA_Vnp *versus* RQA_Vp) of 2.1Å. However, the complexes show little difference in overall MHC structure (rmsd 0.75Å). In RQA_Vnp, S4 adopts a low conformation in the binding groove forming no interactions with surrounding residues (Figure 4D, right), but as for PKD2, phosphorylation results in a raising of the main chain at this position, permitting extensive phosphate-mediated contacts to HLA-A2 (Figure 4D, left). Also similar to PKD2, the resulting conformational change is focussed on the central region of the RQA_V epitope likely to be most critical for T cell recognition (Figure 4B–4D), with substantial differences in both main chain position and individual side chain orientations at residues 4, 5 and 7, frequently sites of TCR contact (Figure 4C). As a result, the molecular surface presented for T cell recognition by RQA_Vp (Figure 4E, left) and RQA_Vnp (Figure 4E, right) epitopes is substantially different.

Comparison of RQA_Vp and RQA_Vnp structures also explains their equivalent affinities for HLA-A2. Although RQA_Vnp lacks any S4-mediated MHC contacts, the low main chain position allows additional side chain interactions and intra-peptide contacts, which help stabilise the complex (Figure 4F, Supplementary Table 2). Upon phosphorylation these are lost due to the phosphate-induced elevation of the position 4 Cα, which results in a more raised peptide conformation (Figure 4F). Therefore, the conformational change permits energetically rich phosphate-mediated contacts to the MHC, but this is balanced by loss of interactions specific for the unmodified peptide conformation, resulting in similar pMHC binding affinities.

### The RQI epitope is preconfigured for phosphate-dependent interaction with MHC

**Figure 5:**
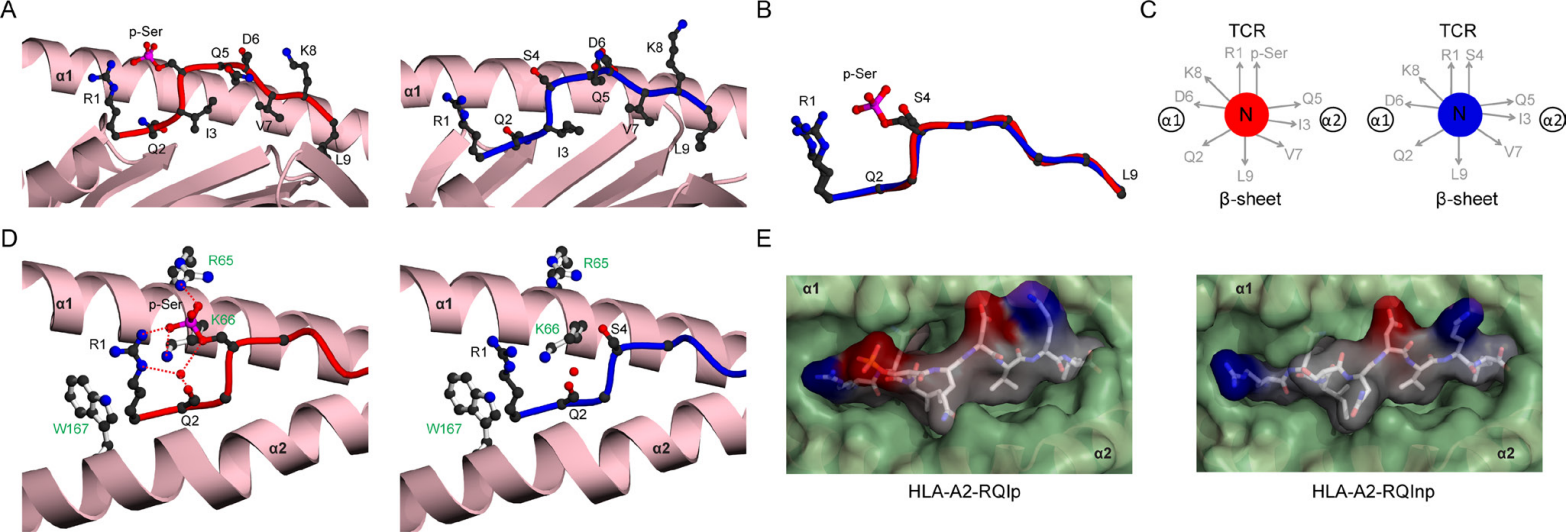
Structural comparison of HLA-A2 bound RQI phosphopeptide in modified and unmodified states (**A**) HLA-A2 bound structures of RQIp (left, red) and RQInp (right, blue), derived from adenosine monophosphate deaminase. The α1-α2 antigen binding platform is shown in ribbon representation (pink), with α2 helix residues 137-166 omitted for clarity. (**B**) Superposition of the RQI main chain structures for phosphorylated (red) and non-phosphorylated (blue) peptides, including the R1 and S4/p-Ser side-chains. (**C**) Side-chain orientation for phosphorylated (left, red) and non-phosphorylated (right, blue) RQI peptides, as viewed along the long axis of the peptide from its N-terminus. (**D**) Interactions of position 4 side chains for phosphorylated (left, red main chain) and non-phosphorylated (right, blue main chain) peptides with HLA-A2 α1-α2 helices (pink). Hydrogen bonds are indicated by red dashed lines; red spheres represent water molecules; for clarity the underlying β-sheet is omitted. HLA-A2 side chains are shown as white sticks and labelled green. (**E**) Molecular surface of phosphorylated (left) and non-phosphorylated (right) RQI peptides, as viewed from the perspective of the TCR. Color scheme as in Figure 4D.

A third canonical phosphopeptide (RQI) was solved in phosphorylated (RQIp) and non-phosphorylated (RQInp) forms in complex with HLA-A2, to 1.7Å (HLA-A2-RQIp) and 2.1Å (HLA-A2-RQInp) respectively (Supplementary Table 1, Figure 5). RQIp exhibits a 75-fold enhanced binding to HLA-A2 relative to RQInp (Kd 25.5 nM versus 1925 nM, respectively). As with previous structures there was little difference observed in overall MHC structure (rmsd 0.62Å) for both complexes. However, unlike PKD2 and RQA_V epitopes (Figure 3, Figure 4), RQIp and RQInp peptides showed no conformational rearrangement (rmsd 0.39Å, Figure 5A–5C), although RQIp exhibited phosphate-mediated contacts (Figure 5D, left) similar to those of other canonical phosphopeptides [15]. As a result, the molecular surfaces of RQIp (Figure 5E, left) and RQInp (Figure 5E, right) accessible for recognition are very similar. Therefore the RQInp peptide closely mimics the main chain conformation of its phosphorylated counterpart, suggesting that, other than the phosphate moiety, the antigenic features exposed to the TCR would be very similar for RQIp and RQInp (Figure 5E).

**Figure 6:**
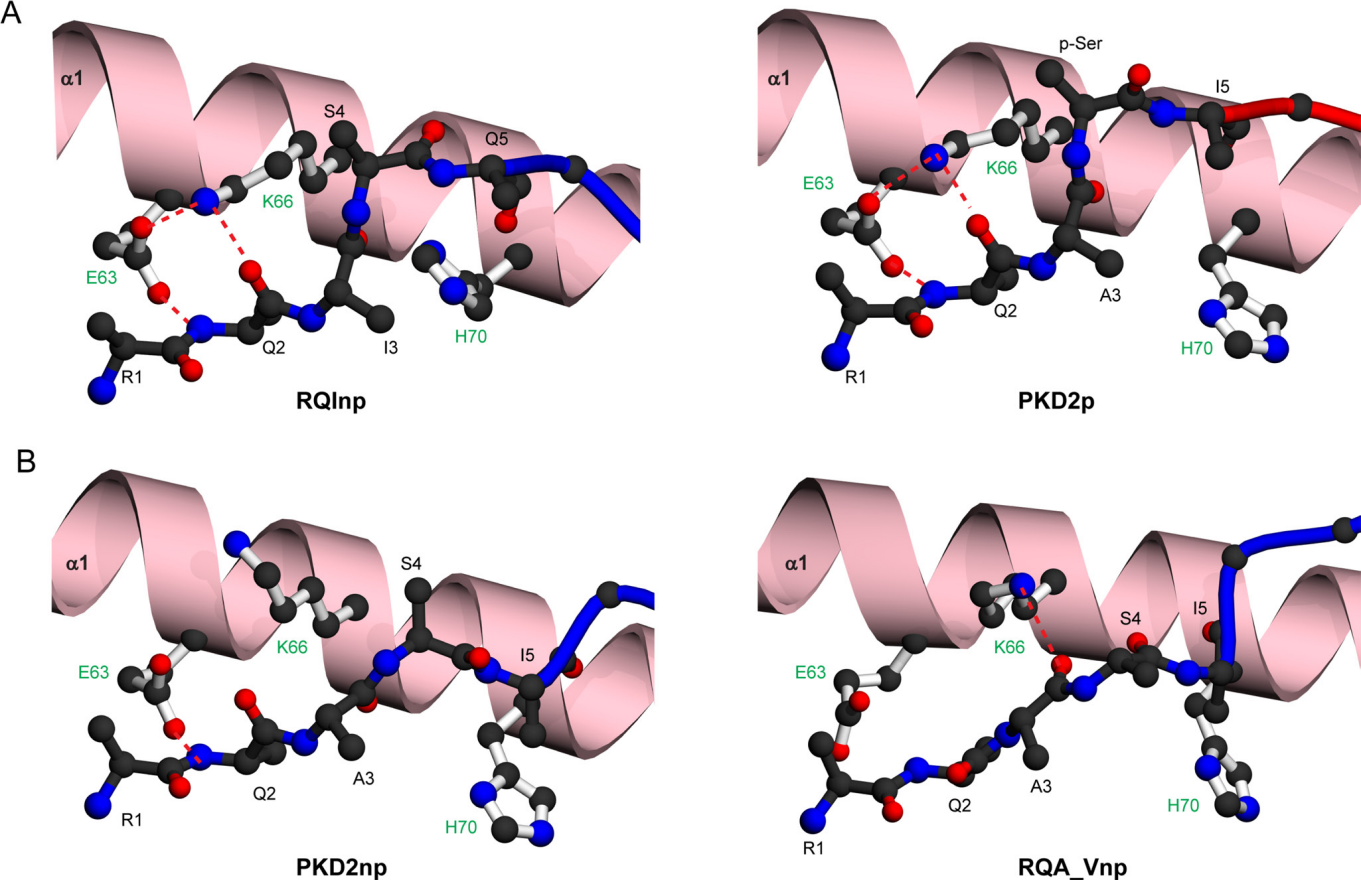
Molecular contacts stabilising the main chain conformations of RQI, PKD2 and RQA_V epitopes (**A**) Comparison of H70 orientation and main chain hydrogen bonding pattern around position 2 of RQInp (left) and PKD2p (right). (**B**) Comparison of H70 orientation and main chain hydrogen bonding pattern around position 2 of PKD2np (left) and RQA_Vp (right). The α1 helix is shown in ribbon representation (pink), with both the α2 helix and the β-sheet omitted for clarity. Hydrogen bonds are indicated by red dashed lines. HLA-A2 side chains are shown as white sticks and labelled green.

Two key reasons underlie these observations. Firstly, in HLA-A2-RQInp, residues E63 and K66 from the α1-helix interact with one another and form a cooperative stabilising hydrogen-bonding network with the peptide backbone amide and carbonyl groups of Q2 (Figure 6A, left). This arrangement is conserved in all phosphopeptide structures (Figure 6A, right), but is disrupted in the unmodified forms of PKD2 (Figure 6B, left) and RQA_V (Figure 6B, right). Secondly, in HLA-A2-RQIp, as for PKD2p (Figure 6A, right) the phosphate-mediated contacts elevate the main chain conformation around positions 4–5. Although these are lost in RQInp, an elevated main chain conformation around position 4 is stabilised critically by H70, which protrudes from the base of the α1 helix and reorientates relative to its position in the phosphopeptide structure, enabling stabilising contacts to the backbone carbonyl of peptide residues at positions 3 and 5 (Figure 6A, left), These two key features allow the RQInp peptide to mimic the main chain conformation of its phosphorylated counterpart, and suggest that, other than the phosphate moiety, the antigenic features exposed to the TCR would be very similar for RQIp and RQInp (Figure 5).

### TCR binding to a human CDC25b-derived phosphopeptide is highly phosphate dependent and epitope-specific

Having established that phosphorylation has diverse effects on epitope structure, we aimed to test whether a phosphorylated epitope could be distinguished from its unmodified counterpart by the TCR. We previously generated phosphopeptide-specific CD8^+^ T cells by immunising mice expressing a transgenic recombinant HLA-A*0201 molecule (AAD) with activated bone-marrow-derived dendritic cells pulsed with synthetic phosphopeptides corresponding to those naturally presented on the surface of human tumour cell lines [5]. Despite the ability to isolate functional phosphopeptide specific T-cells, attempts to generate clonal populations of RQA_V specific T-cells for TCR isolation or produce soluble TCRs of sufficient quality from T-cell clones specific for PKD and RQI were unsuccessful. However we were able to generate soluble TCR (termed TCRpCDC25b) using cDNA sequences isolated from a T cell clone that recognised a phosphopeptide epitope derived from the CDC25b protein (GLLGpSPVRA, subsequently termed GLLGpS) of sufficient quality for surface plasmon resonance analysis. Previous structural analyses have indicated that the GLLGpS epitope undergoes only very minor alterations in main chain conformation [12], suggesting it would provide a relatively demanding test of phosphate-dependent recognition by the TCR.

**Figure 7:**
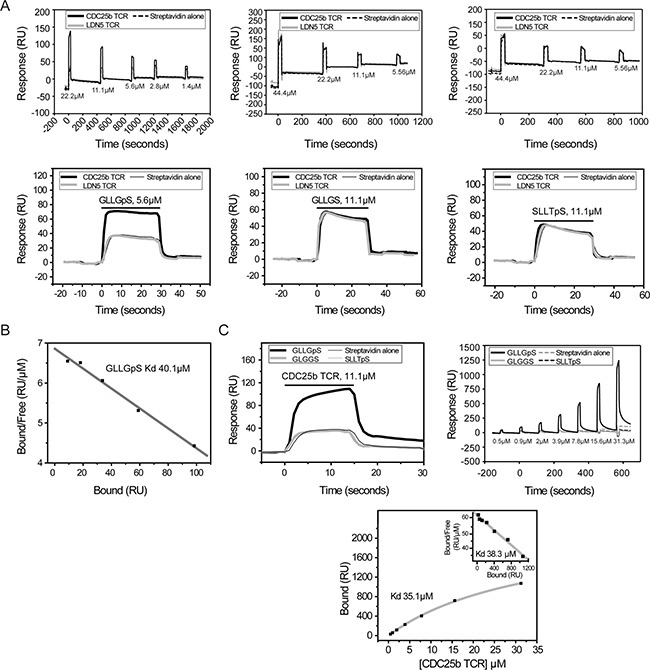
Epitope-specific and phosphate-dependent recognition of HLA-A2-GLLGpS (**A**) Injection of HLA-A2-GLLGpS at different concentrations indicated specific binding to CDC25b-specific TCR (left panel), whereas neither injection of HLA-A2-GLLGS (middle panel) nor HLA-A2-SLLTpS (right panel) resulted in specific binding. (**B**) Scatchard analysis of HLA-A2-GLLGpS binding to immobilised CDC25b-specific TCR. (**C**) Specific binding of CDC25b-specific TCR to immobilised HLA-A2-GLLGpS (left panel). Injection of CDC25b-specific TCR at different concentrations (right panel). Equilibrium binding analysis of CDC25b-specific TCR binding to immobilised HLA-A2-GLLGpS (bottom panel, with Scatchard analysis shown inset). Equilibrium binding analyses were carried out twice independently, once in either orientation, with comparable Kd values (35–41 μM, average 38.7 μM +/− 2.7 μM) obtained from both Scatchard analyses and hyperbolic fitting to saturation binding plots.

Injection of HLA-A2-GLLGpS complex over immobilised TCRpCDC25b yielded substantially higher responses than over control surfaces (LDN5 and streptavidin), indicating specific binding (Figure 7A, left). In contrast, injection of HLA-A2 containing GLLGS, lacking the phosphorylation at position 5, resulted in equivalent responses over immobilised TCRpCDC25b and control surfaces (Figure 7A, middle), indicating that recognition of GLLGpS by TCRpCDC25b was entirely dependent on the presence of the phosphate. To investigate if recognition was dependent on the GLLGS amino acid sequence, we also tested binding of immobilised TCRpCDC25b to HLA-A2 containing a different phosphopeptide featuring a p-Ser at position 5 (SLLTpSPPKA (termed SLLTpS) derived from Thyroid hormone receptor interacting protein 12) [5]. Injection of HLA-A2-SLLTpS complex yielded identical responses over TCRpCDC25b and control surfaces (Figure 7A, right), indicating recognition of GLLGpS by TCRpCDC25b was not only phosphate-dependent but also peptide sequence-specific. We then measured the strength of TCRpCDC25b phosphopeptide recognition by equilibrium affinity analysis, repeating injections of HLA-A2-GLLGpS over a range of concentrations (Figure 7B). These experiments indicated an affinity (Kd) of ˜40.1 μM (Figure 7B). Similar injections and equilibrium binding analysis in the opposite orientation, confirmed both the specificity and affinity (Kd ˜35.1 μM) of the interaction (Figure 7C). In both orientations, the presence of low-level protein aggregates was evident at higher concentrations. These studies suggest phosphate-dependent, epitope-specific recognition is consistent with TCR/pMHC affinities that are comparable in strength to conventional TCR/pMHC interactions [21] involving non-phosphorylated antigens.

## DISCUSSION

Phosphopeptides are emerging as an important group of MHC-associated antigens that may be of particular relevance in the context of cancer. Consistent with dysregulation of kinase pathways in cancer, our recent work in the context of haematological malignancies identified numerous phosphopeptide species selectively presented on tumour tissue but absent on matched normal tissue, and also indicated that in many cases the source proteins for such tumour-associated phosphopeptides are encoded by oncogenes strongly linked to leukemogenesis, highlighting their potential as immunotherapeutic targets [11]. Furthermore, whereas robust CD8^+^ immunity against many such phosphopeptides was observed in healthy individuals, immunity was impaired in leukaemia patients, particular those with a poor prognosis. These findings implicate immunity to phosphopeptide antigens in tumour immunosurveillance, and highlight their potential importance in future cancer immunotherapy strategies such as tumour vaccines and T cell adoptive transfer approaches. In this context, gaining a solid understanding of the potential molecular effects of phosphorylation on epitope conformation and TCR interaction is a priority, and could impact on the choice of targeting strategy.

Our study establishes that phosphorylation can have radical effects on the antigenic identity of MHC-bound peptides. We show that phosphorylation can have major effects on peptide conformation, and outline the structural basis of this effect. For both PKD2 and RQA_V epitopes, a similar structural mechanism was involved. Interestingly, despite this, the overall consequences for pMHC affinity were clearly highly context dependent, and emphasise the benefit of parallel structural and pMHC affinity analyses to fully understand the effects of phosphorylation on individual epitopes. We and others have previously established the molecular “ground rules” for canonical phosphopeptide presentation by MHC molecules based on extensive structural, peptide-MHC affinity and mutagenesis approaches [12, 15]. These studies demonstrated that the phosphate moiety is an integral part of the epitope, mediating energetically significant contacts to positively charged MHC residues, and explained how phosphorylation can substantially enhance the binding of peptides that exhibit low affinities for MHC. Importantly, for the current set of canonical phosphopeptide antigens presented in this study, the phosphate mediated contacts to the MHC are highly conserved with previously determined canonical phosphopeptide-HLA-A2 structures, suggesting that the same molecular “ground rules” are likely to apply, particularly with respect to the energetics of the phosphate mediated stabilisation of the MHC.

For PKD2, the conformational change resulted in a net increase in pMHC contacts, explaining the overall phosphate-dependent increase in affinity. In contrast, for RQA_V, while the conformational change results in new stabilising contacts to HLA-A2, this was at the expense of multiple contacts only present in the unmodified form, and hence the net affinity was unchanged. However, in both cases, the resulting changes in main chain conformation were focussed on the central region of each epitope and thus would be expected to have critical effects on TCR recognition [20, 22]. Consistent with this, Cobbold *et al* generated T cells that recognise the RQA_V epitope in both a phosphate-dependent and epitope-specific manner [11]. Phosphorylated epitopes such as these cannot therefore be regarded as merely conventional peptide epitopes with “cherries on top”. Rather, in terms of their conformation a subset of phosphopeptides are completely novel. Such “conformational neoantigens” may be particularly likely to occur within the canonical phosphopeptide repertoire, since the mechanism underlying epitope rearrangement appears to be driven by the phosphorylation at position 4, which introduces novel contacts to the class I MHC [15]. This mechanism may operate for many other canonical phosphopeptides.

Secondly, in contrast to the situation above, many phosphopeptides, including other canonical epitopes, may naturally adopt conformations in the unmodified state that (upon phosphorylation at position 4) allow classic phosphate-mediated contacts, and consequently no/little conformational change would be expected upon modification. The proportion of canonical phosphopeptides in this category is unclear, but this group includes both RQIpS, and the RVApS phosphopeptide derived from Insulin Receptor Substrate 2 [5, 12]. Moreover, many non-canonical phosphopeptides may remain conformationally unaltered.

Our surface plasmon resonance binding studies, the first analysis of TCR interaction with phosphopeptide-MHC complexes, build on these findings. They establish that the ability of T cells to recognise phosphopeptide-MHC molecules in both an epitope-specific and phosphate-dependent manner can reside within the TCR itself, and that TCR discrimination between modified/unmodified forms can be essentially complete. Moreover, our studies highlight the presence of the phosphate moiety can be sufficient to enable such modification-dependent and antigen-specific discrimination even for epitopes in which phosphorylation-induced conformational alterations are minimal, as for the GLLGpS epitope we focussed on. The finding that a single phosphorylation can make such a dramatic difference to the biophysics of TCR/pMHC interaction provides hope that even in the absence of conformational change, altered phosphorylation, for example during different stages of oncogenesis, has a profound effect on antigenic identity and may be sufficient to break T cell tolerance, thereby inducing post-translational-modification-dependent immune responses. In addition, previous studies have highlighted that pCDC25b-specific CD8^+^ T lymphocytes displayed effector functions against target cells pulsed with epitopes corresponding to the phosphorylated forms of the antigen but not the non-phosphorylated equivalent [5]. Our findings that the pCDC25b-specific TCR bound the phosphorylated but not the non-phosphorylated form of the CDC25b peptide suggests the explanation for these data lie in direct TCR-based discrimination of phosphorylation status within the context of antigen-specific recognition.

Therefore in summary, our results highlight that the effects of phosphorylation on epitope structure are diverse. For individual peptides, such as the canonical RQA_V epitope studied here, these can include radical changes in peptide conformation, including in central regions likely to influence TCR recognition. Consistent with this, T cells specific for the RQA_V peptide isolated from healthy donors were found to distinguish the phosphorylated from the non-phosphorylated form of the epitope [11]. For other peptides, the effects of phosphorylation on conformation may be minimal. In such cases, the impact of such moieties on T cell recognition may significantly depend on the position of the modification. Our results show that for a central P5 modification (the non-canonical epitope GLLG), phosphorylation can result in TCR binding that is both epitope-specific and phosphate-dependent, consistent with previous T cell studies [5]. Notably, canonical P4 phosphorylations are also oriented towards the central P5 position. However, given that TCR recognition is typically focussed substantially on such central peptide residues, it is likely that, in the absence of phosphorylation-induced changes in epitope conformation, phosphorylations at extreme ends of the peptide may often be ignored during T cell recognition.

Our results therefore provide a basis for understanding phosphopeptide-specific immune responses observed in healthy individuals and cancer patients [11], and provide additional impetus for therapeutic targeting of phosphopeptides as candidate antigens for cancer immunotherapy. Recent studies have highlighted mutated cancer neoantigens as a target for potent anti-tumour immune responses [23], particularly for tumours/tumour subtypes with high mutational burden (e.g. melanoma, lung). However, for the many tumours and tumour subtypes with relatively low mutational burden, alternative antigenic targets may be required. Given the widespread oncogene-driven dysregulation of kinase pathways in such low mutation groups, therapeutic targeting of the cancer phenotype *via* tumour-associated phosphopeptide antigens remains an attractive alternative possibility.

In general, such targeting strategies should specifically target the phosphorylated form since these are likely to be upregulated on transformed cells, whereas unmodified counterparts may be present on normal untransformed cells. Our findings that such phosphorylated forms are highly antigenically distinct from their unmodified counterparts establish a molecular basis for antigen-specific targeting of such phosphorylated forms, for example employing either vaccination [14] or adoptive T cell transfer [13]. In principle, phosphorylation-induced conformational neoantigens may be particularly attractive targets, as their altered conformation could circumvent central tolerance, potentially increasing the size of the T cell repertoire responding to vaccination. In comparison, the T cell repertoire capable of recognising phosphopeptides unaltered in conformation by their modification may be somewhat narrower due to T cell tolerance; potentially favouring TCR gene transfer approaches employing highly selected TCRs that display phosphate-dependent recognition. In this context, the RQA_V and PKD2 phosphopeptides we describe are attractive targets for vaccination and TCR gene transfer approaches. Notably, not only does RQA_V exhibit a dramatic phosphorylation-induced conformational rearrangement, but we have recently detected presentation of HLA-A2-RQA_Vp on primary tumour samples from a range of human leukemias and were able to generate human T cells recognising RQA_V in an epitope-specific and phosphate-dependent manner (10). In combination, these features highlight conformationally unique phosphopeptides such as RQA_V as compelling candidates for cancer immunotherapy approaches.

## MATERIALS AND METHODS

### Class I MHC production and crystallisation

HLA-A2 heavy chain and β2-microglobulin were expressed in *E. coli*, purified from inclusion bodies and refolded together with synthetic phosphopeptide or unmodified equivalent, and purified by gel filtration, as described [24]. Crystallization conditions were identified by vapor-diffusion with a mosquito nanolitre crystallization robot (TTP Labtech) at 22 °C. Conditions tested included the Index (Hampton Research), Wizard (Emerald Biosystems) and JCSG^+^ (Molecular Dimensions) screens at concentrations of 10–20 mg/ml. Favorable conditions were optimized on a larger scale (Supplementary Table 1) and yielded diffraction-quality crystals that typically grew to 200 mm × 200 mm × 100 mm after 3–4 days. Crystals of HLA-A2 in complex with phosphorylated/non-phosphorylated forms of PKD2 were grown as described [12].

### Crystallisation of HLA-A2-RQA_Vnp in complex with LILRB1

For crystallization of the HLA-A2-RQA_Vnp complex in complex with LILRB1, recombinant LILRB1 D1D2 was expressed in *E. coli*, purified from inclusion bodies, refolded and purified by gel filtration as described [19]. Crystallization conditions for the LILRB1-HLA-A2-RQA_Vnp complex were identified as above, using purified HLA-A2-RQA_Vnp mixed with LILRB1 in a 1:1 ratio at 10.5 mg/ml. The most favourable condition, 18% PEG 3350, 0.2M ammonium acetate and 0.1M HEPES pH 7.4, yielded diffraction-quality crystals that grew to ˜ 300 mm × 200 mm × 200 mm after 2–3 weeks.

### Peptide-MHC data collection, structure solution and refinement

HLA-A2-peptide complex crystals were soaked in reservoir buffer containing increasing concentrations (5%, 10% and 15% (v/v)) of ethylene glycol or glycerol before being ‘flash-cooled’ at 100K in a nitrogen gas stream (Oxford Cryosystems). X-ray data were collected to 1.6–2.7Å on an “in house” MicroMax 007HF microfocus rotating anode X-ray generator (Rigaku) with a Saturn CCD detector. Data sets were integrated, scaled and merged with the XDS suite [25]. HLA-A2-peptide complex structures were determined by molecular replacement with MOLREP [26] using as the search model a previously determined HLA-A2 structure with peptide residues omitted. The LILRB1-HLA-A2-RQA_Vnp complex structure was solved by molecular replacement with CNS [27] using LILRB1-HLA-A2 complex as the search model [19].

Molecular-replacement calculations yielded unambiguous rotation and translation function solutions. The molecular models were refined with CNS [27] and REFMAC5 [26]. Refinement progress was verified by monitoring the R_free_ value [28]. Models were subjected to alternating simulated annealing and positional refinement followed by isotropic B factor refinement. Electron-density maps showed unbiased features in the electron density (full sequence of each peptide), confirming the validity of the molecular replacement solution. Model manipulations were performed with COOT [29]. Once the R factor values were below 30%, water molecules were included if they appeared in Fo – Fc maps contoured at over 3σ and were within hydrogen-bonding distance to chemically acceptable groups. The final data processing and refinement statistics are listed in Supplementary Table 1. The quality of the final refined models was verified with PROCHECK [26] and WHATCHECK [30]. Most residues were well defined in all structures, except for a few solvent-exposed side chains. Hydrogen bonding, hydrophobic and van der Waals contacts were analyzed with CONTACT (CCP4) [26]. Structural figures were produced with Pymol (http://www.pymol.org), or with the POVScript program [31] and rendered with the Persistence of Vision Raytracer (http://www.povray.org), with molecular surfaces generated using DelPhi [32].

### Peptide-MHC affinity assays

HLA-A2 heavy chain was expressed in *E. coli*, refolded with β_2_M and the peptide NLVPMVATV, and purified as described above. Competitive peptide binding assays were carried out as described [33]. Test peptide concentrations covered a 100,000-fold range, with each concentration assayed in triplicate. MHC-peptide complexes were captured on microplates coated with monoclonal antibody W6/32 (to human HLA) and washed, and radioactivity quantified with a microscintillation counter. The concentration of test peptide that displaced 50% of the radiolabeled peptide (IC50) was calculated. In these conditions (in which the concentration of the label is less than the concentration of MHC and the IC50 is greater than or equal to the concentration of MHC), the IC50 is a reasonable approximation of the dissociation constant [34].

### TCR/peptide-MHC binding assays

Experiments utilised a BIAcore 3000 and HBS-EP buffer, at a flow rate of 10 μl.min^−1^. For HLA-peptide injections, recombinant TCRs were produced in the *Drosophila* expression system incorporating C-terminal biotinylation tags, biotinylated *in vitro* using BirA, and immobilised to streptavidin-coated CM5 surfaces. In the reverse orientation, purified *Drosophila*-expressed CDC25b-specific TCR was injected over streptavidin-coated surfaces to which biotinylated HLA-peptide complexes (either HLA-A2-GLLGpS or control complexes, incorporating C-terminal biotinylation tags) were immobilised. Data were analysed using BIAevaluation 3.1 and Origin graphing software.

### Accession numbers

Atomic coordinates and structure factors are deposited in the Protein Data Bank under accession numbers 4NNX (HLA-A2-PKD2p), 4NNY (HLA-A2-PKD2np), 4NO2 (HLA-A2 RQA_Vp), 4NO0 (HLA-A2-RQA_Vnp), 4NO3 (HLA-A2-RQIp) and 4NO5 (HLA-A2-RQInp).

## SUPPLEMENTARY MATERIALS TABLES



## References

[1] Depontieu FR, Qian J, Zarling AL, McMiller TL, Salay TM, Norris A, English AM, Shabanowitz J, Engelhard VH, Hunt DF, Topalian SL (2009). Identification of tumor-associated, MHC class II-restricted phosphopeptides as targets for immunotherapy. Proc Natl Acad Sci USA.

[2] Li Y, Depontieu FR, Sidney J, Salay TM, Engelhard VH, Hunt DF, Sette A, Topalian SL, Mariuzza RA (2010). Structural basis for the presentation of tumor-associated MHC class II-restricted phosphopeptides to CD4+ T cells. J Mol Biol.

[3] Meyer VS, Drews O, Gunder M, Hennenlotter J, Rammensee HG, Stevanovic S (2009). Identification of natural MHC class II presented phosphopeptides and tumor-derived MHC class I phospholigands. J Proteome Res.

[4] Zarling AL, Ficarro SB, White FM, Shabanowitz J, Hunt DF, Engelhard VH (2000). Phosphorylated peptides are naturally processed and presented by major histocompatibility complex class I molecules *in vivo*. J Exp Med.

[5] Zarling AL, Polefrone JM, Evans AM, Mikesh LM, Shabanowitz J, Lewis ST, Engelhard VH, Hunt DF (2006). Identification of class I MHC-associated phosphopeptides as targets for cancer immunotherapy. Proc Natl Acad Sci USA.

[6] Blume-Jensen P, Hunter T (2001). Oncogenic kinase signalling. Nature.

[7] Evan GI, Vousden KH (2001). Proliferation, cell cycle and apoptosis in cancer. Nature.

[8] Greenman C, Stephens P, Smith R, Dalgliesh GL, Hunter C, Bignell G, Davies H, Teague J, Butler A, Stevens C, Edkins S, O’Meara S, Vastrik I (2007). Patterns of somatic mutation in human cancer genomes. Nature.

[9] Haluska FG, Tsao H, Wu H, Haluska FS, Lazar A, Goel V (2006). Genetic Alterations in Signaling Pathways in Melanoma. Clin Cancer Res.

[10] Oka M, Kikkawa U (2005). Protein kinase C in melanoma. Cancer Metast Rev.

[11] Cobbold M, De La Pena H, Norris A, Polefrone JM, Qian J, English AM, Cummings KL, Penny S, Turner JE, Cottine J, Abelin JG, Malaker SA, Zarling AL (2013). MHC class I-associated phosphopeptides are the targets of memory-like immunity in leukemia. Sci Transl Med.

[12] Petersen J, Wurzbacher SJ, Williamson NA, Ramarathinam SH, Reid HH, Nair AK, Zhao AY, Nastovska R, Rudge G, Rossjohn J, Purcell AW (2009). Phosphorylated self-peptides alter human leukocyte antigen class I-restricted antigen presentation and generate tumor-specific epitopes. Proc Natl Acad Sci USA.

[13] Dudley ME, Rosenberg SA (2003). Adoptive-cell-transfer therapy for the treatment of patients with cancer. Nat Rev Cancer.

[14] Melief CJ (2008). Cancer immunotherapy by dendritic cells. Immunity.

[15] Mohammed F, Cobbold M, Zarling AL, Salim M, Barrett-Wilt GA, Shabanowitz J, Hunt DF, Engelhard VH, Willcox BE (2008). Phosphorylation-dependent interaction between antigenic peptides and MHC class I: a molecular basis for the presentation of transformed self. Nat Immunol.

[16] LaValle CR, George KM, Sharlow ER, Lazo JS, Wipf P, Wang QJ (2010). Protein kinase D as a potential new target for cancer therapy. Biochim Biophys Acta.

[17] Marafioti T, Mancini C, Ascani S, Sabattini E, Zinzani PL, Pozzobon M, Pulford K, Falini B, Jaffe ES, Muller-Hermelink HK, Mason DY, Pileri SA (2004). Leukocyte-specific phosphoprotein-1 and PU.1: two useful markers for distinguishing T-cell-rich B-cell lymphoma from lymphocyte-predominant Hodgkin's disease. Haematologica.

[18] Anderson KJ, Allen RL (2009). Regulation of T-cell immunity by leucocyte immunoglobulin-like receptors: innate immune receptors for self on antigen-presenting cells. Immunology.

[19] Willcox BE, Thomas LM, Bjorkman PJ (2003). Crystal structure of HLA-A2 bound to LIR-1, a host and viral major histocompatibility complex receptor. Nat Immunol.

[20] Madden DR, Garboczi DN, Wiley DC (1993). The antigenic identity of peptide-MHC complexes: a comparison of the conformations of five viral peptides presented by HLA-A2. Cell.

[21] Davis MM, Boniface JJ, Reich Z, Lyons D, Hampl J, Arden B, Chien Y (1998). Ligand recognition by alpha beta T cell receptors. Annu Rev Immunol.

[22] van der Merwe PA, Davis SJ (2003). Molecular interactions mediating T cell antigen recognition. Annu Rev Immunol.

[23] Schumacher TN, Schreiber RD (2015). Neoantigens in cancer immunotherapy. Science.

[24] Garboczi DN, Hung DT, Wiley DC (1992). HLA-A2-peptide complexes: refolding and crystallization of molecules expressed in Escherichia coli and complexed with single antigenic peptides. Proc Natl Acad Sci USA.

[25] Kabsch W (1993). Automatic processing of rotation diffraction data from crystals of initially unknown symmetry and cell constants. J Appl Crystallogr.

[26] CCP4 (1994). The CCP4 suite: programs for protein crystallography. Acta Crystallogr D Biol Crystallogr.

[27] Brunger AT (2007). Version 1.2 of the Crystallography and NMR system. Nat Protoc.

[28] Brunger AT (1992). Free R value: a novel statistical quantity for assessing the accuracy of crystal structures. Nature.

[29] Emsley P, Cowtan K (2004). Coot: model-building tools for molecular graphics. Acta Crystallogr D Biol Crystallogr.

[30] Hooft RW, Vriend G, Sander C, Abola EE (1996). Errors in protein structures. Nature.

[31] Fenn TD, Ringe D, Petsko GA (2003). POVScript+: a program for model and data visualization using persistence of vision ray-tracing. J Appl Crystallogr.

[32] Rocchia W, Alexov E, Honig B (2001). Extending the applicability of the non-linear Poisson-Boltzmann equation: multiple dielectric constants and multivalent ions. J Phys Chem B.

[33] Sidney J, Southwood S, Mann DL, Fernandez-Vina MA, Newman MJ, Sette A (2001). Majority of peptides binding HLA-A*0201 with high affinity crossreact with other A2-supertype molecules. Hum Immunol.

[34] Gulukota K, Sidney J, Sette A, DeLisi C (1997). Two complementary methods for predicting peptides binding major histocompatibility complex molecules. J Mol Biol.

