# Supplementary material for: The antigenic identity of human class I MHC phosphopeptides is critically dependent upon phosphorylation status
Source: Oncotarget. 2017 Apr 8;8(33):54160–72. doi: 10.18632/oncotarget.16952 (PMC5589570; doi:10.18632/oncotarget.16952)
Supplement: Supplementary file 1 [file oncotarget-08-54160-s001.pdf]

The antigenic identity of human class I MHC phosphopeptides is critically dependent upon phosphorylation status

Supplementary Materials

Supplementary Table 1: Crystallisation, data processing and refinement statistics

	PKD2		RQA_V		RQI	
**PDB ID CODE**	4NNX	4NNY	4NO2	4NO0	4NO3	4NO5
Peptide sequence	RQApSLSISV	RQASLSISV	RQApSIELPSMAV	RQASIELPSMAV	RQIpSQDVKL	RQISQDVKL
Peptide source	PKD2	PKD2	LSP-1	LSP-1	AMPD2	AMPD2
**Crystallisation**						
Precipitant (%)	PEG 3350 (11)	PEG 3350 (15)	PEG 8K (20)	PEG 3350 (18)	PEG 3350 (12)	PEG 3350 (19)
Buffer (0.1 M)	HEPES pH 7.5	HEPES pH 7.5	HEPES pH 7.5	HEPES pH 7.5	Tacsimate pH 8	Bis Tris pH 6.5
Salt (M)	NaCl (0.15)	NaCl (0.2)	-	NH4-Acet (0.2)	-	Na tartrate (0.1)
Additive (0.003 M)	$MgCl_2$, $CdCl_2$	$MgCl_2$, $CdCl_2$	-	-	-	-
Protein (mg/ml)	10	10	17	10.5	11	11
**Data Processing**						
Resolution range (Å)	20–2.1	20–1.9	20–2	20–2.7	20–1.7	20–2.1
Unit cell Parameters						
a , b, c (Å)	61.1, 79.8, 111.3	60.4, 79.8, 110.7	119.2, 54.7, 75.2	117.3, 117.3, 96	118, 54.2, 75.1	56.4, 79.3, 56.8
α, β, γ (°)	90, 90, 90	90, 90, 90	90, 104.8, 90	90, 90, 120	90, 104.9, 90	90, 115.5, 90
Space Group	$P2_12_12_1$	$P2_12_12_1$	C2	P3121	C2	$P2_1$
Total reflections	228414	346902	317693	773823	334999	299127
Unique reflections	31648	42471	31594	21363	46573	26307
Multiplicity	7.2	8.2	10	36	7.2	11.4
Completeness (%)[a]	99 (96.4)	99 (94.6)	98.9 (96.1)	98.9 (99.5)	91.9 (75.4)	99.3 (98.8)
R_{merge} (%)[b]	10.8 (38.5)	9.2 (52.9)	5.4 (46.4)	12.6 (81.6)	3.4 (32.8)	8.8 (24.0)
I/σ(I)	15.3 (4.8)	16.6 (4.9)	31.6 (4.5)	33.2 (6.2)	34 (4.7)	25.2 (11.4)
**Refinement**						
Resolution (Å)	20–2.1	20–1.9	20–2	20–2.7	20–1.7	20–2.1
Reflections used	31643	42469	31330	21363	44256	25030
R_{cryst} (%)[c]	19.8	20.8	20.8	26.9	20.8	18.8
R_{free} (%)[d]	23.6	22.8	24.9	30.9	23.2	23.7
Protein residues	382	382	386	580	383	382
Water molecules	211	301	258	51	391	266
RMS deviations						
Bond lengths (Å)	0.016	0.009	0.010	0.018	0.0082	0.0096
Bond angles (°)	1.46	1.07	1.17	1.63	1.11	1.14

Figures in parentheses in the data processing section apply to data in the highest resolution shell. [a]Completeness = (number of independent reflections/total theoretical number). [b]R_{merge} (I) = (Σ|I(i) - <I(h)>|/ΣI(i)), where I(i) is the ith observation of the intensity of the hkl reflection and <I> is the mean intensity from multiple measurements of the h,k,l reflection. [c]R_{cryst} (F) = $\Sigma_h||F_{obs}(h)| - |F_{calc}(h)||/\Sigma h|F_{obs}(h)|$ and $|F_{calc}(h)|$ are the observed and calculated structure factor amplitudes for the h, k, l reflection. [d]R_{free} is calculated over reflections in a test set not included in atomic refinement (5% of data). Peptides are derived from PKD2 (protein kinase D2), LSP-1 (lymphocyte specific protein 1) and AMPD2 (adenosine monophosphate deaminase 2).

Supplementary Table 2: Comparison of phosphate-independent stabilising contacts mediated by RQA_Vp and RQA_Vnp peptides and HLA-A*0201

	RQA_Vp	RQA_Vnp
**Peptide**	**HLA-A2**	
R1	Tyr159 (3)	Tyr159 (3)
Q2	Tyr159 (5)	Tyr159 (6)<br>**Q2 (O) - Y159 (OH) 2.8Å**
A3	Tyr159 (14)	Tyr159 (8)
S4/p-Ser	(See Table S3)	-
I5	Gln155 (11)<br>Leu156 (5)<br>Intra M10 (1)	His70 (21)<br>Thr73 (3)<br>**I5 (O) - His70 (N) 2.9Å**<br>Arg97 (7)<br>Tyr99 (1)<br>Intra M10 (3)
E6	-	Ala69 (5)<br>Thr73 (4)
Total contacts	39	61

Values in parentheses correspond to the number of hydrophobic and van der Waals contacts mediated between the peptide and HLA-A2. Hydrogen bonds with donor to receptor distances of 2.3-3.4Å are shown in bold. Intra corresponds to an intra-peptide stabilising interaction between I5 and M10.
